# Stacking *AsFMT* overexpression with *BdPMT* loss of function enhances monolignol ferulate production in *Brachypodium distachyon*

**DOI:** 10.1111/pbi.13606

**Published:** 2021-05-15

**Authors:** Rebecca A. Smith, Cynthia L. Cass, Deborah L. Petrik, Dharshana Padmakshan, John Ralph, John C. Sedbrook, Steven D. Karlen

**Affiliations:** ^1^ U.S. Department of Energy Great Lakes Bioenergy Research Center and the Department of Biochemistry Wisconsin Energy Institute University of Wisconsin Madison WI USA; ^2^ School of Biological Sciences Illinois State University Normal IL USA; ^3^ U.S. Department of Energy Great Lakes Bioenergy Research Center Madison WI USA; ^4^ Department of Biology Northeastern State University Tahlequah OK USA

**Keywords:** BAHD acyltransferase, lignin acylation, DFRC method, bioenergy, bioengineering, biomass, plant biotechnology

## Abstract

To what degree can the lignin subunits in a monocot be derived from monolignol ferulate (ML‐FA) conjugates? This simple question comes with a complex set of variables. Three potential requirements for optimizing ML‐FA production are as follows: (1) The presence of an active FERULOYL‐CoA MONOLIGNOL TRANSFERASE (FMT) enzyme throughout monolignol production; (2) Suppression or elimination of enzymatic pathways competing for monolignols and intermediates during lignin biosynthesis; and (3) Exclusion of alternative phenolic compounds that participate in lignification. A 16‐fold increase in lignin‐bound ML‐FA incorporation was observed by introducing an *AsFMT* gene into *Brachypodium distachyon*. On its own, knocking out the native *p*‐*COUMAROYL‐CoA MONOLIGNOL TRANSFERASE* *(BdPMT*) pathway that competes for monolignols and the *p*‐coumaroyl‐CoA intermediate did not change ML‐FA incorporation, nor did partial loss of *CINNAMOYL‐CoA REDUCTASE1* (*CCR1*) function, which reduced metabolic flux to monolignols. However, stacking *AsFMT* into the *Bdpmt‐1* mutant resulted in a 32‐fold increase in ML‐FA incorporation into lignin over the wild‐type level.

## Introduction

Lignins are heterogeneous polymers composed from an array of building blocks, primarily the canonical monolignols sinapyl, coniferyl and *p*‐coumaryl alcohols, that give rise to the S, G and H units in the polymer. The plasticity of lignification results in the inclusion of varying amounts of these monolignols along with monolignol conjugates and other phenolic compounds (Mottiar *et al*., 2016; Sederoff *et al*., 1999; Vanholme *et al*., 2019). The production of monolignol conjugates is the result of the activity of a specific class of BAHD acyltransferases known as monolignol transferases. These enzymes form ester conjugates between monolignols and acyl‐CoA substrates prior to their incorporation into the growing lignin polymer chain (Ralph, 2010). Since the discovery of monolignol ferulates (ML‐FA) (Wilkerson *et al*., 2014), and the subsequent quantification of native ML‐FA levels in the lignins of many plant species across the angiosperms (Karlen *et al*., 2016), there have been several strategies tested to increase the compositional percentage of these units in plant lignins. The seminal ML‐FA work introduced the dicotyledonous *Angelica sinensis FERULOYL‐CoA MONOLIGNOL TRANSFERASE* (*FMT*) gene into hybrid‐poplar (*Populus alba* × *grandidentata*), driven by either the ubiquitous *35S* or the xylem‐specific *CesA8* promoter (Wilkerson *et al*., 2014). This strategy successfully increased ML‐FA by an estimated sevenfold (Wilkerson *et al*., 2014). Subsequently, ML‐FA incorporation into lignin in rice (*Oryza sativa*) was increased by upregulating the native *FMT* *(OsAT5* or *OsFMT)* gene using a ubiquitin promoter or mutant activation tagging. The resulting *OsFMT* over‐expressing rice lines produced an estimated fivefold increase in ML‐FA (Karlen *et al*., 2016). Perhaps the most striking example was in Arabidopsis, a plant that has to date never shown any evidence of native ML‐FA production, nor the production of any other monolignol conjugates, in which the introduction of *AsFMT* resulted in the production and subsequent incorporation of ML‐FA into the lignin (Smith *et al*., 2017b).

Early in the development of plant lines with higher ML‐FA lignins, it was logical to surmise that monolignol transferases could compete for substrates (Sibout *et al*., 2016; Withers *et al*., 2012), and therefore monolignol transferases that make other monolignol conjugates would need to be suppressed to introduce and increase the production of ML‐FA. However, as noted above, that did not seem to be the case in poplar in which the introduction of *AsFMT* alone was sufficient to increase ML‐FA levels (˜7‐fold), or in rice in which upregulation of the native *Os*FMT enzyme also increased ML‐FA levels (˜5‐fold). This hypothesis was further brought into question in *Brachypodium distachyon* (Brachypodium) with the complete knockout of *p‐COUMAROYL‐CoA MONOLIGNOL TRANSFERASE* (*PMT*) through mutagenesis (Petrik *et al*., 2014). Knocking out *BdPMT* had a twofold effect: (1) It removed a pathway competing for both monolignols (H, G and S) and *p*‐coumaroyl‐CoA (*p*CA‐CoA, an upstream compound in the biosynthesis of FA‐CoA and monolignols); and (2) It removed ML‐*p*CA from the monomer pool, eliminating a compound that participates in lignification and thus reducing the compositional complexity of the lignin. The *Bdpmt‐1* knockout mutant line had negligible amounts of ML‐*p*CA (Petrik *et al*., 2014) but, even though there was a putative acyltransferase with FMT activity still present, based on the presence of ML‐FAs, there was not a corresponding increase of ML‐FA in the lignin (Karlen *et al*., 2016). This result suggested that any native FMT in Brachypodium has limited activity, and/or is spatially and/or temporally separated from the increased amounts of substrates in the *Bdpmt‐1* mutant. In addition, *p*‐coumaroyl‐CoA is a branch‐point between the lignin and flavonoid biosynthetic pathways. Loss of BdPMT activity may therefore increase the flux of *p*CA‐CoA into the synthesis of flavonoids, such as cell‐wall‐bound tricin, thus presenting another competitive pathway to the production of ML‐FA.

**Figure 1:**
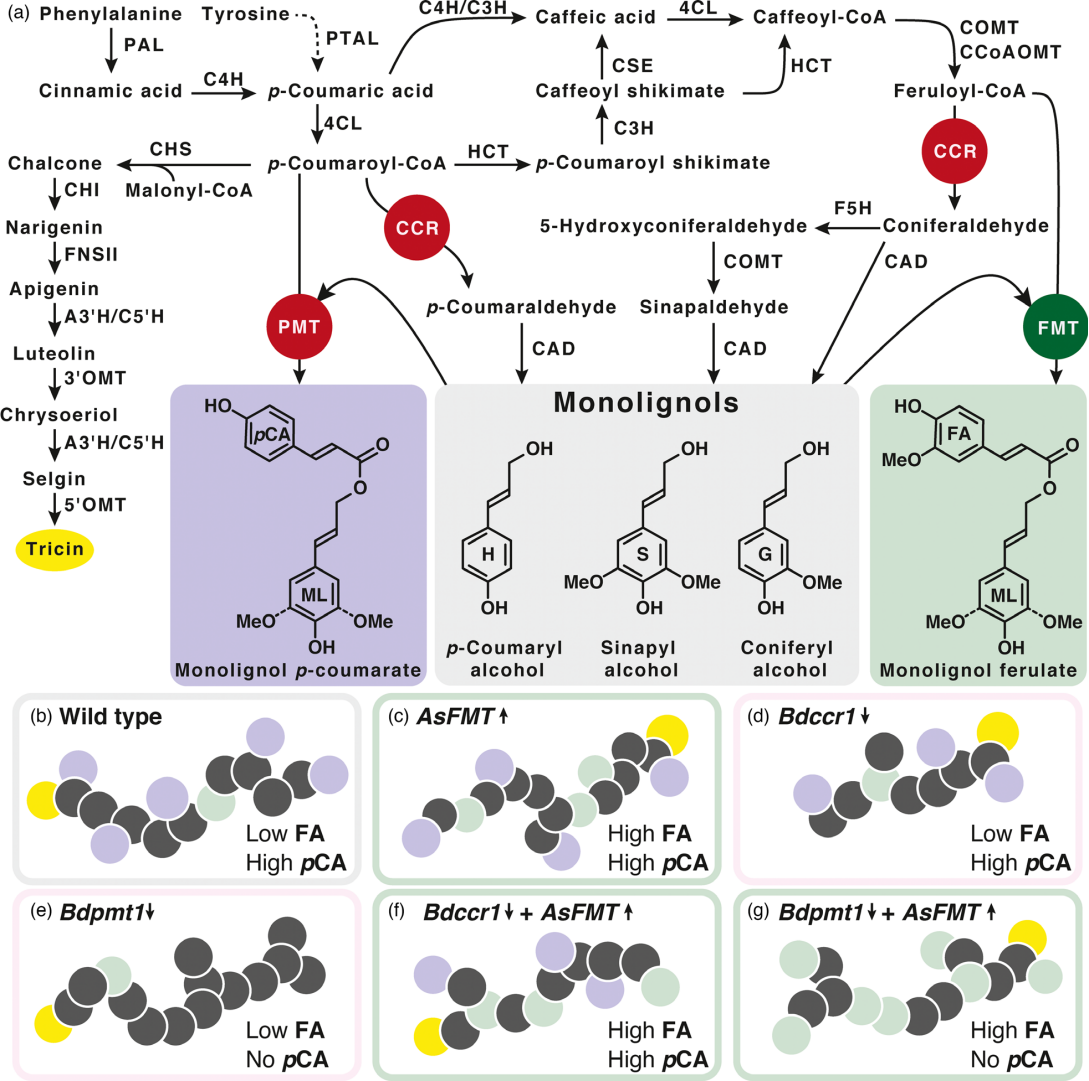
(a) Lignin and tricin biosynthetic pathways for Brachypodium. Enzymes highlighted in red were knocked out or their expression was suppressed. Enzymes highlighted in green were over‐expressed. (b–g) Grey circles = monolignols (S, G, and H), purple circles = ML‐*p*CA, green circles = ML‐FA, yellow circles = tricin (chain initiation sites). (b) The lignin in wild‐type plants. (c) Overexpression of *AsFMT* increases ML‐FA incorporation. (d) Reducing the expression of *Bdccr1‐1* reduces ML production resulting in lower total lignin. (e) Knocking out *Bdpmt‐1* eliminates ML‐*p*CA production and reduces the lignin compositional complexity. (f) Crossing *Bdccr1‐1* with *AsFMT* over‐expression lines might increase ML‐FA incorporation. (g) Crossing *Bdpmt‐1* knockout with *AsFMT* over‐expression lines substantially increases ML‐FA incorporation and reduces the lignin compositional complexity.

Because removing the ML‐*p*CA competitive pathway alone did not increase the amount of ML‐FA in Brachypodium lignins, we hypothesized that increasing the pool of available FA‐CoA by instead introducing a bottleneck into the lignin biosynthetic pathway just below FA‐CoA would increase ML‐FA amounts. This was tested in maize (*Zea mays*) by targeted suppression of *CINNAMOYL‐CoA REDUCTASE1* (*CCR1*), which encodes a key enzyme in the monolignol biosynthetic pathway (Figure 1), resulting in a reduction of total lignin content and a threefold to fivefold increase in the level of lignin‐incorporated ML‐FA (Smith *et al*., 2017a).

We selected *Brachypodium distachyon* as a model plant to probe the interplay between building up the pool of FA‐CoA accessible to FMT enzymes and the abundance of FMT enzymes. We stacked the traits from three different strategies: (1) Overexpression of *AsFMT* using *Zea mays* ubiquitin‐1 promoter to ensure the presence of an active FMT; (2) Suppression of the *BdCCR1* gene, a strategy that was successful in *Zea mays*; and (3) Utilization of the *Bdpmt‐1* mutant as a background line to remove a monolignol transferase pathway that competes for *p*CA‐CoA and monolignols (Figure 1). These three strategies were then stacked (*AsFMT* × *Bdpmt‐1* and *AsFMT* × *Bdccr1‐1*) to elucidate how their interactions altered lignin‐incorporated ML‐FA and determine the bioenergy‐relevant changes in stem tissue digestibility.

## Results

*As*FMT was introduced into Brachypodium wild‐type plants to determine whether higher levels of FMT enzyme yielded higher levels of ML‐FA. To assist in the visual identification of transgenic plants expressing *AsFMT*, constructs were made fusing the *ENHANCED YELLOW FLUORESCENT PROTEIN* (*EYFP*) gene in‐frame to either the N‐ or C‐ terminus of the *AsFMT* gene (*EYFP:AsFMT* and *AsFMT:EYFP*, respectively). These binary vector constructs were stably introduced into Brachypodium by *Agrobacterium*‐mediated tissue‐culture transformation followed by plant regeneration. Plants transgenic for the *AsFMT:EYFP* construct fluoresced at much higher levels than those harbouring *EYFP:AsFMT*. Fluorescence from *AsFMT:EYFP* plants was easily detectable in various organs in both 10‐day‐old seedlings and in plants 31 days after planting (Figure S1A–D), indicating that the *AsFMT:EYFP* gene product was translated into a full‐length AsFMT‐EYFP fusion. This was confirmed by Western blot analysis, probing culm tissue protein extracts with an anti‐YFP antibody to determine that plants expressing both *AsFMT:EYFP* and *EYFP:AsFMT* produced the predicted band sizes for full‐length fusion proteins (Figure S1E,F). Moreover, the relative intensity levels of EYFP‐derived fluorescence for the *AsFMT:EYFP* and *EYFP:AsFMT* transgenic plant lines correlated well with the amounts of fusion protein detected by Western blot analysis. Therefore, we focused our studies on the highest fluorescing *AsFMT:EYFP* plant lines and used them for all further experiments.

**Figure 2:**
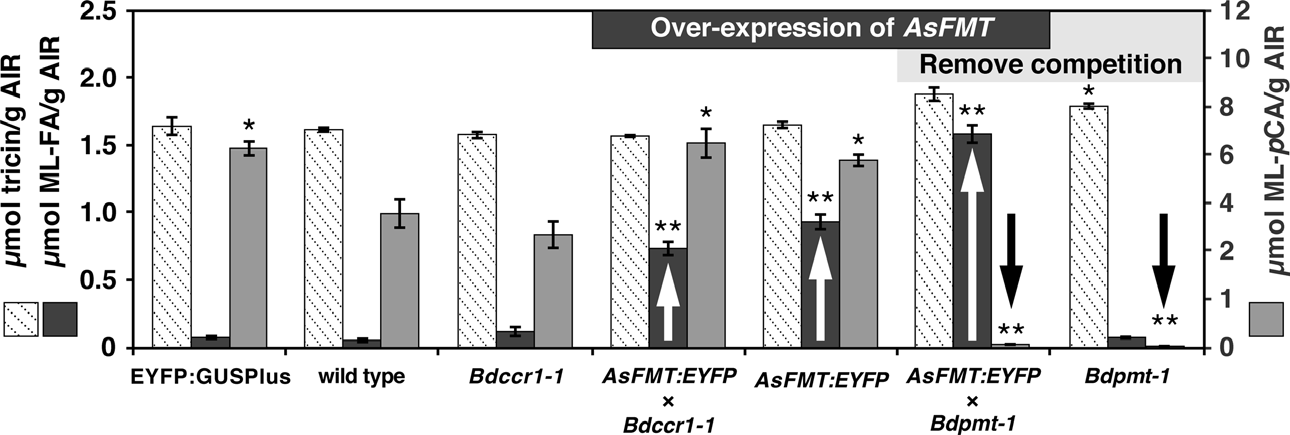
A comparison of cell‐wall‐bound monolignol conjugates as quantified by the DFRC assay on μmol/g AIR basis. The amount of ML‐FA (dark grey) detected increased with the introduction of *AsFMT* and further in the trait‐stacked *AsFMT:YFP* × *Bdpmt‐1* hybrid plants. The competing lignin monomer ML‐*p*CA (light grey) dropped to trace levels in both the knockout mutant *Bdpmt‐1* and the trait‐stacked *AsFMT:YFP* × *Bdpmt‐1* hybrid plants. The alternative biosynthesis pathway for *p*CA‐CoA derived product tricin (hashed bars) increased a small amount in the *Bdpmt‐1* mutant. Bars represent SEM for *n* = 3 biological replicates. Significant differences from wild type, as determined by the Student’s *t*‐test, are indicated with * for *Ρ* < 0.05 and ** for *Ρ* < 0.01.

*AsFMT:EYFP* plants were morphologically indistinguishable from wild type (Figure S2A, B) and contained similar amounts of acetyl‐bromide‐soluble lignin (ABSL) (Table 1). To determine whether changes had occurred in lignin‐bound monolignol conjugates, derivatization followed by reductive cleavage (DFRC) lignin compositional analysis was performed on alcohol‐insoluble residue (AIR), prepared by solvent extraction of senesced culm tissue. The DFRC assay cleaves β‐aryl ether bonds in lignin but leaves esters fully intact, releasing diagnostic lignin‐derived conjugates (*i.e*. sinapyl 7,8‐dihydro‐*p*‐coumarate and sinapyl 7,8‐dihydroferulate; S‐DH*p*CA and S‐DHFA, respectively) (Lu and Ralph, 1999; Regner *et al*., 2018). DFRC showed as much as a 16‐fold increase of ML‐FA in *AsFMT*:*EYFP*‐expressing plant culms versus wild type (Figure 2; Table 1). For example, in the highest‐expressing *AsFMT:EYFP* line, ˜0.8 μmol ML‐FA could be released per gram AIR compared to 0.05 μmol/g AIR for wild type. The Student’s *t*‐test indicated that the increase in quantified ML‐FA was statistically significant (*P* < 0.001). The amount of ML‐*p*CA was significantly higher in the *AsFMT*:*EYFP* line compared to wild type, but not the GUS control line. As the amount of ML‐*p*CA was not significantly different from the amount of ML‐*p*CA observed in the *YFP‐GUS* control lines, we hypothesized that this difference might be attributed to natural ML‐*p*CA variation between lines, as has been observed in other studies (Karlen *et al*., 2020).

**Table 1:** The lignin components released from extract‐free whole cell wall culm tissues and quantified by DFRC. Compounds reported are the monomers that are in the lignin and are set to equal the acetylated/hydrogenated compounds detected by GC‐MRM‐MS. Significant differences from wild type, as determined by the Student’s *t*‐test, are indicated with * for *Ρ* < 0.05 and ** for *Ρ* < 0.01. †The *Bdpmt‐1* mutant line originated from a different wild‐type background and was previously shown to be significantly different from the associated wild‐type plants (Petrik *et al*., 2014)

Compound	*EYFP:GUSPlus*	Wild type	*Bdccr1‐1*	*AsFMT:EYFP* × *Bdccr1‐1*	*AsFMT:EYFP*	*AsFMT:EYFP* × *Bdpmt‐1*	*Bdpmt‐1*
DFRC	μmol/g AIR ± SEM	μmol/g AIR ± SEM	μmol/g AIR ± SEM	μmol/g AIR ± SEM	μmol/g AIR ± SEM	μmol/g AIR ± SEM	μmol/g AIR ± SEM
G‐*p*CA	0.01 ± 0.00^*^	0.01 ± 0.00	0.01 ± 0.00	0.02 ± 0.00	0.02 ± 0.01^*^	*trace* ^**^	*trace* ^**^
G‐FA	*trace*	*trace*	*trace*	0.01 ± 0.00	0.01 ± 0.01	0.10 ± 0.02^*^	*trace*
S‐*p*CA	5.9 ± 0.4	4.0 ± 0.8	3.3 ± 0.8	6.0 ± 0.9	6.1 ± 0.2	0.2 ± 0.0^**^	0.1 ± 0.0^**^
S‐FA	0.1 ± 0.0	0.0 ± 0.0	0.1 ± 0.1	0.7 ± 0.1	0.8 ± 0.1^**^	3.1 ± 0.5^**^	0.1 ± 0.0
tricin	1.64 ± 0.13	1.62 ± 0.03	1.58 ± 0.05	1.57 ± 0.01	1.65 ± 0.05	1.88 ± 0.10	1.79 ± 0.04^*^
Acyl bromide soluble lignin (ABSL)							
wt% (g/g AIR)	22.3 ± 1.0	23.3 ± 1.8	22 ± 0.6	24 ± 0.7	20.5 ± 2.1	19.9 ± 1.6	18.9 ± 0.3^*†^

To determine if increasing the amount of substrate available to the native Brachypodium FMT increased the production of ML‐FA, the *Bdccr1‐1* T‐DNA mutant allele was isolated and studied (Figure S3A). As noted above with maize (Smith *et al*., 2017a), and with other *ccr* mutants (Derikvand *et al*., 2008; Jones *et al*., 2001; Leplé *et al*., 2007; Tamasloukht *et al*., 2011), the Brachypodium *Bdccr1‐1* mutant homozygous for the T‐DNA insertion had a reduction in *ccr1* gene expression (Figure S3B). Homozygous *Bdccr1‐1*mutant plants appeared phenotypically wild type and had normal growth (Figure S3C), which may be due to the fact that the lesion results in only a partial loss of function. However, compared to wild‐type Brachypodium, these mutants still displayed a significant decrease in Klason lignin content (Figure S4A), the expected reduction in thioacidolysis‐quantified syringyl (S), guaiacyl (G), and *p*‐hydroxyphenyl (H) units (Figure S4B), and lighter coloration with phloroglucinol‐HCl staining indicative of reduced lignin hydroxycinnamaldehyde end‐groups (Figure S4C,D). DFRC analysis of the *Bdccr1‐1* Brachypodium plants revealed that neither the ML‐FA nor the ML‐*p*CA levels were significantly different than in wild type (Figure 2; Table 1).

To probe the size/availability of the FA‐CoA pool for increased production of ML‐FAs, the *AsFMT* gene was introduced into the *Bdccr1‐1* background. This was accomplished by cross‐pollination of *AsFMT:EYFP* plants into the *Bdccr1‐1* background. Of the resulting F_2_‐generation progeny, those that were genetically *AsFMT:EYFP* × *Bdccr1‐1* homozygous exhibited ML‐FA levels that were significantly higher than in wild type (0.73 μmol/g AIR versus 0.05 μmol/g AIR; Figure 2; Table 1). This increase in ML‐FA corresponds to the same change observed in the parent *AsFMT:EYFP* line, indicating that the presence of AsFMT alone was most likely responsible for the increase in ML‐FA.

*Bd*PMT and *As*FMT require the same monolignol substrates, and *Bd*PMT esterifies *p*‐coumaroyl‐CoA which would otherwise be converted downstream into both of the *As*FMT substrates, feruloyl‐CoA and monolignols. We have shown previously that the *Bdpmt‐1* mutation alone does not alter the level ML‐FAs in the culm tissue (Karlen *et al*., 2016) (Figure 2; Table 1). Therefore, we cross‐pollinated the *Bdpmt‐1* mutant (Petrik *et al*., 2014) with *AsFMT:EYFP* plants to remove a competing acyltransferase pathway and ensure the presence of an active FMT enzyme throughout lignification. DFRC analysis of the F_2_‐generation progeny expressing *AsFMT:EYFP* and homozygous for the *Bdpmt‐1* mutation (*AsFMT:EYFP* × *Bdpmt‐1*) showed a 32‐fold increase in the level of ML‐FAs over wild‐type plants (Figure 2; Table 1). This level is significantly higher than either parent plant line expressing *AsFMT:EYFP* or containing the *Bdpmt‐1* mutation (Figure 2; Table 1).

As with the *AsFMT:EYFP* plants, the *AsFMT:EYFP* × *Bdpmt‐1* plants were morphologically indistinguishable from wild type (Figure S2C). The trait‐stacked *AsFMT:EYFP* × *Bdpmt‐1* plants also contained amounts of ABSL similar to wild type (Table 1). These results suggest that this genetic approach enhanced lignin ML‐FA levels at the expense of the competing ML‐*p*CA conjugates and generated plants that otherwise were nearly indistinguishable from wild type.

The lignin biosynthetic pathway is part of a larger phenylpropanoid metabolic array. The flavonoid biosynthetic pathway, for example, branches off from the lignin biosynthetic pathway at *p*CA‐CoA (Figure 1). There is therefore potential competition between the production of flavonoids and monolignol conjugates. One flavonoid of interest in this study was the cell wall‐bound flavonoid tricin that, due to its association with lignin, is found in the same spatial and temporal location as lignin and monolignol conjugate biosynthesis (Lan *et al*., 2015). To test whether the flavonoid pathway was altered by the different metabolic fluxes in the lignin biosynthetic mutants and crosses, we quantified the amount of tricin in the DFRC samples. The addition of *AsFMT* and/or the loss of BdCCR activity did not impact tricin production (Figure 2, Table 1). However, the *Bdpmt‐1* mutant did have significantly higher levels of tricin than wild‐type Brachypodium (Figure 2, Table 1). This suggests that some of the pooled *p*CA‐CoA in the mutant background is diverted to the flavonoid biosynthetic pathway and the production of tricin. The *AsFMT:EYFP* × *Bdpmt‐1* lines also displayed an increase in cell wall‐bound tricin, but it is not statistically significant compared to the wild‐type lines. It is therefore possible that the AsFMT enzyme, which is more efficient at ML‐FA production than the native Brachypodium FMT, can compete with the flavonoid biosynthetic pathway to draw the pool of *p*CA‐CoA back into monolignol conjugate production.

**Figure 3:**
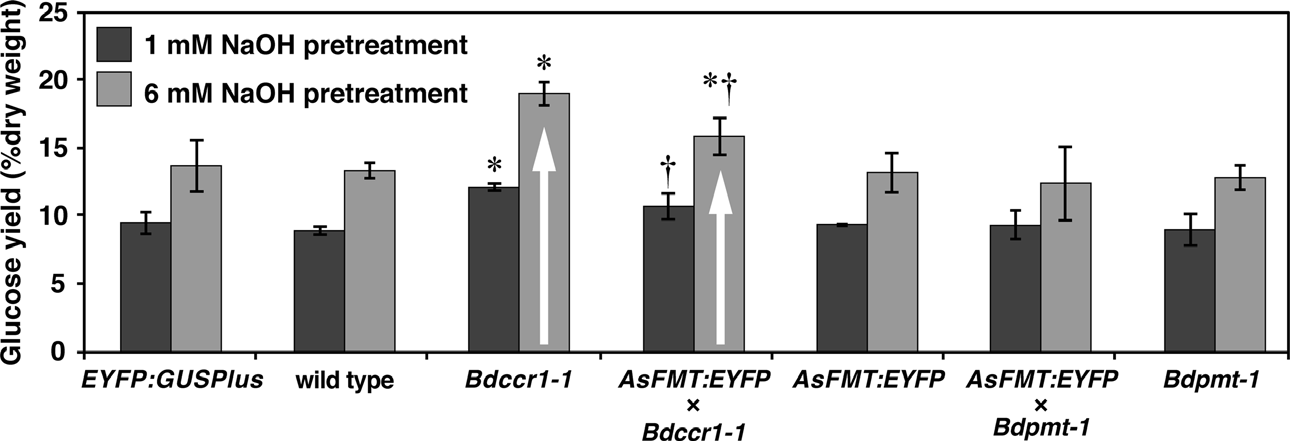
Glucose released from partial saccharification analysis following 1 mm or 6 mm NaOH pretreatment. Bars represent means ± SEM for *n* = 3 biological replicates. Student’s *t*‐test determined significant differences from wild type are indicated with * for *Ρ* < 0.05 and the significant differences between *Bdccr1‐1* and *AsFMT:EYFP* × *Bdccr1‐1* with † for *Ρ* < 0.05.

All the transgenic lines generated in this study were ground, pretreated with either 1 mM or 6 mM NaOH at 30 °C, and then subjected to a partial saccharification analysis. *AsFMT:EYFP* had a slight, but not statistically significant, increase in glucose release when treated with 1 mm NaOH compared to wild‐type plants (Figure 3). *Bdccr1‐1* and *AsFMT:EYFP* × *Bdccr1‐1* plants clearly had significantly higher glucose release than wild‐type Brachypodium under either pretreatment (Figure 3). The glucose release from *AsFMT:EYFP* × *Bdccr1‐1* plants was significantly lower than that of the *Bdccr1‐1* single mutant. Neither the *Bdpmt‐1* nor the *AsFMT:EYFP* × *Bdpmt‐1* homozygous plants had improved glucose release. Combined, the order of digestibility (least to most recalcitrant) was found to be *Bdccr1‐1* > *AsFMT:EYFP* × *Bdccr1‐1* > *AsFMT:EYFP* ≈ *Bdpmt‐1* ≈ *AsFMT:EYFP* × *Bdpmt‐1* ≈ wild type.

## Discussion

Since the discovery of monolignol conjugates, we have tried to determine the limit for incorporation of a single type of γ‐substituted monolignol conjugate (*e.g*. ML‐*p*CA or ML‐FA) into the cell wall. In nature, there are several indications that levels can be higher than we typically see, nearing 100% acylated monolignol complements to lignification. For example, the level of monolignol acetates in kenaf was over 50% (Ralph, 1996), and as much as 80% in abaca (del Río *et al*., 2008). More recently, a seagrass, *Posidonia oceanica*, has been shown to have lignins that are particularly highly *p*‐hydroxybenzoylated (Rencoret *et al*., 2020). Overexpression of *FMT* or *PMT* genes alone in both eudicot and monocot species was able to achieve significant increases in lignin‐incorporated ML‐FA or ML‐*p*CA. In some cases, this introduced monolignol conjugates into plants in which they are not natively found. However, obtaining high levels of monolignol conjugates is not just an issue of having enough enzyme, but is also a function of substrate availability and competing pathways. We therefore set out to produce Brachypodium plants with an active FMT enzyme throughout secondary cell wall formation using a ubiquitin promoter (to increase ML‐FA production), build up a pool of FA‐CoA substrate, and eliminate the PMT enzyme competing for substrates.

We chose to introduce and express *AsFMT* rather than overexpress the native Brachypodium (*BdFMT*) or the closely related rice (*Oryza sativa*) *OsFMT* to avoid any potential sense‐suppression phenomena. The eudicot *AsFMT* shares low sequence homologies with the monocot *OsFMT*, suggestive of convergent evolution (Wilkerson *et al*., 2014). Brachypodium expressed the *AsFMT:EYFP* fusion protein as a functional enzyme capable of significantly increasing ML‐FA production and subsequent incorporation into lignins (Figure 2). The DFRC analytical method determined that ML‐FA incorporation into the lignin had increased 16‐fold over the wild‐type control plants. This definitively determined that the highly divergent *As*FMT was functional in a grass species and that its substrates (monolignols and FA‐CoA) were temporally and spatially available. The increase in ML‐FA production did not appear to occur at the expense of tricin biosynthesis; the utilization of available FA‐CoA did not change the amount of cell‐wall‐bound tricin as quantified by the DFRC analysis (Figure 2, Table 1).

Assuming that the availability of monolignols is not the issue, we attempted to repeat the strategy of suppressing CCR1 to increase ML‐FA incorporation (Smith *et al*., 2017a). Contrary to our results from the *Zea mays Zmccr1* mutant, Brachypodium *Bdccr1‐1* mutants did not produce any more ML‐FA than the wild‐type plants. However, the *Bdccr1‐1* mutants did reduce the total Klason lignin content indicating that the CCR activity was significantly reduced. This indicates that either the *Bdccr1‐1* partial loss of *BdCCR1* production did not build up as much FA‐CoA or the native FMT enzyme could not take advantage of the increase in FA‐CoA. Again, there was also no change in the amount of cell‐wall‐bound tricin as quantified by the DFRC analysis (Figure 2, Table 1), indicating that, if there was an increase in available FA‐CoA, it was not temporally and/or spatially available to be rerouted into tricin biosynthesis.

Compared to other species in the Poaceae family (sorghum, switchgrass, maize, wheat, etc.), the native levels of ML‐FA in Brachypodium are very low (Karlen *et al*., 2016). Although a *Bd*FMT enzyme has yet to be identified, we hypothesize that it might be much less efficient than previously identified FMTs, such as *As*FMT and *Os*FMT, or at least expressed at much lower levels. Because the *As*FMT‐expressing plants successfully increased ML‐FAs by 16‐fold, we crossed them with the *Bdccr1‐1* mutant to provide an FMT enzyme capable of utilizing excess FA‐CoA and monolignols. The *AsFMT:EYFP* × *Bdccr1‐1* homozygous plants had levels of ML‐FA similar to *AsFMT:EYFP* lines. Therefore, the issue of increasing ML‐FA in the *Bdccr1‐1* lines was not an issue of active FMT enzyme, but rather that the suppression of *CCR* activity was not strong enough or other biosynthetic pathways were outcompeting *As*FMT for substrate.

One of the enzymes that competes with FMT for substrates from the lignin biosynthetic pathway is PMT, which forms ML‐*p*CA from *p*CA‐CoA and monolignol substrates (Figure 1). In the knockout mutant *Bdpmt‐1*, the production of ML‐*p*CA drops to trace levels, simplifying the chemical diversity of the lignin and removing the chemical demand for both monolignols and *p*CA‐CoA. As noted, although ML‐*p*CA content of the lignin was low, there was not an associated increase in ML‐FA incorporation. There was, however, a slight increase in tricin (Figure 2, Table 1). These data indicate that, at least in Brachypodium, the low native levels of ML‐FA were not due to competition with *Bd*PMT for substrate. This further supports the hypothesis that the limiting factor in ML‐FA production might be the temporal and/or spatial availability of active *Bd*FMT enzyme or its low enzyme activity.

Stacking *AsFMT:EYFP* expression with the *Bdpmt‐1* knockout mutant would therefore provide the missing active enzyme and probe how the full complement of our strategy played out. The result of this genetic stack was a 32‐fold increase in ML‐FA incorporation from wild type, which was 1.9‐fold higher than with *AsFMT:EYFP* expression alone. This demonstrates that, when enzyme activity/enzyme abundance is not a factor, these FMT and PMT enzymes likely compete for substrate. The stacked‐trait plants notably recovered to wild‐type levels of ABSL, either due to the increased lignin‐bound FA, which also absorbs at λ = 280 nm, restoration of the native flux of the lignin biosynthetic pathway (removing inhibitory effects of excess *p*CA‐CoA), or through another mechanism.

To determine a target goal for the increase of ML‐FA from the wild‐type levels, we need to determine a theoretical limit of ML‐FA incorporation into lignin. One method would be to compare the differences in quantified ML‐*p*CAs and ML‐FAs (combined ML‐conjugates) between the *AsFMT:YFP* × *Bdpmt‐1*, *AsFMT:EYFP* and wild‐type plants on a μmol/g AIR basis. The amount of releasable ML‐conjugates in the wild‐type plants was 4.02 μmol/g AIR; 3.97 μmol/g AIR of which was ML‐*p*CA. The *AsFMT:EYFP* plants had 6.93 μmol/g AIR releasable ML‐conjugates, with increases in both ML‐FAs (0.84 μmol/g AIR) and ML‐*p*CA (6.09 μmol/g AIR, up 2.12 μmol/g AIR from the wild type). The stacked‐trait *AsFMT:EYFP* × *Bdpmt‐1* plants decreased in releasable ML‐conjugates (1.67 μmol/g AIR). Contrary to the wild‐type plants, the releasable ML‐conjugates in the stacked‐trait plants were almost all ML‐FAs (1.58 μmol/g AIR), masking the virtually complete loss of ML‐*p*CA (down 3.88 μmol/g AIR). Assuming that the level of ML‐conjugates is limited to the 6.93 μmol/g AIR, the amount observed in the *AsFMT:EYF*P line, then theoretically the amount of ML‐FAs in the stacked‐trait *AsFMT:EYFP* × *Bdpmt‐1* could still be increased another 5.26 μmol/g AIR (4.1‐fold) to bring the releasable ML‐FAs up to 9.93 μmol/g AIR. If this was achieved, then the theoretical limit for ML‐FAs in Brachypodium is 139‐fold more than the wild type. In our stacked *AsFMT:EYFP* × *Bdpmt‐1*, we are currently at 32‐fold over wild type, theoretically leaving room to increase ML‐FA levels by another 107‐fold. The implications of this observation are that competing monolignol transferases (*i.e*. PMT) need to be knocked out in order to obtain the highest possible ML‐FA incorporation into lignin.

The main driving force for this research was to determine how the amount of ML‐FAs present in the lignin changes with the addition of an active FMT enzyme and suppression of enzymes that compete for FA‐CoA. The goal is to eventually transfer the technology to commercial crops in which higher amounts of ML‐FA equate to better plants for application in a biorefinery or use as a forage crop. To that end, we probed the digestibility of the biomass using a mild technique that releases only the most accessible sugars. Using the full set of *AsFMT:EYFP*, *Bdccr1‐1*, *Bdpmt‐1*, wild type and the two crossed lines, we were able to also test which strategy impacts digestibility of grasses more; lower lignin content or ML‐FA content. We found that the plant lines with lower lignin content showed significant improvement in digestibility. There was no correlation between ML‐FA or ML‐*p*CA content and digestibility in this study, but it has been observed in other studies and other species (Sibout *et al*., 2016; Wilkerson *et al*., 2014). However, it should be noted that Brachypodium already is considered easily digestible and that even in the best lines, the amount of ML‐FA is still extremely low. Stacking the *CCR1* lignin reduction with the *As*FMT to increase ML‐FAs partially recovers the wild‐type lignin content and, with it, biomass recalcitrance. These results indicate that perhaps, for grasses that have some of the highest native ML‐FA contents, modest increases in ML‐FAs will not provide large, or even significant, improvement in cell‐wall digestibility. This is contrary to what was observed in the hardwood poplar, which has very low native ML‐FA content and is considered much more recalcitrant than the grasses. The changes in observed digestibility of *AsFMT*‐poplar lines were associated with the increased amount of quantified ML‐FA and not with the total lignin (e.g. Line *CesA8::FMT*‐5 had one of the highest total lignin content and was the most digestible) (Wilkerson *et al*., 2014).

The additive effects of *As*FMT with either the *Bdccr1‐1* or *Bdpmt‐1* mutant support the hypothesis that, in order to maximize ML‐FA production and subsequent incorporation into lignin: (1) There must be a highly active FMT enzyme present throughout monolignol production; (2) Enzymatic pathways that compete with the lignin biosynthetic pathway for monolignols and intermediates in the pathway must be suppressed or eliminated; and 3) Alternative phenolic compounds (e.g. ML‐*p*CA) that participate in lignification must be suppressed or eliminated.

## Experimental procedures

### Brachypodium seed handling and plant growth

Seed sterilization, agar plating and transformant selection were as previously described (Cass *et al*., 2015; Petrik *et al*., 2014). Plants were grown in a 50 : 50 mix of Sun Gro Redi‐earth and Metro‐Mix 510 soil in 4‐inch pots in growth chambers (20 h light : 4 h dark photoperiod, 22 °C, 50% humidity). Control plants were either wild‐type Bd21‐3 seedlings plated on non‐selective plates or planted directly in soil, or hygromycin‐selected plants harbouring a *Zea mays* ubiquitin‐1 promoter with its intron driving GUSPlus (Cambia, Canberra, ACT 2601, Australia) in pWBVec8.

### Generation of *AsFMT* constructs

The open reading frame (ORF) clone for *Angelica sinensis Feruloyl‐CoA Monolignol Transferase* (*AsFMT*, JA758320) was provided by Dr. Curtis Wilkerson (Wilkerson *et al*., 2014). The *Zea mays* ubiquitin‐1 promoter:*EYFP:AsFMT* fusion construct was generated by Gateway cloning (Invitrogen, Thermo Fisher Scientific, Waltham, MA USA) the *AsFMT* ORF in pDONR221 into a modified pHWGY2 (Karimi *et al*., 2002) vector in which the 35S promoter had been replaced with the maize ubiquitin‐1 promoter plus intron from pStarling (Christensen and Quail, 1996). The *Zea mays* ubiquitin‐1 promoter::*AsFMT:EYFP* fusion construct was synthesized by PCR‐amplifying the *AsFMT* ORF and removing the stop codon with primers forward 5′‐CCCCGGATCCCATGACGATCATGGAGGTTCAAG‐3′ and reverse 5′‐ CCCCTCGAGGAAGCGAAAGCAGAGATGGTG‐3′, digestion of the PCR product with BamHI and XhoI restriction enzymes (NEB), ligation into pENTR2B, and then Gateway cloning the fragment into the modified pHYWG2 vector. The *Zea mays* ubiqitin‐1 promoter::*EYFP:GUSPlus* control construct was synthesized by amplifying the *GUSPlus* ORF from the pGPro8 vector (Genbank accession JN593327.1) using forward primer 5′‐ GTCGACTGGATCCTATGGTAGATCTGAG‐3′ and reverse primer 5′‐ GAGACTCGAGTCACACGTGATGGTGATGGTG‐3′, digestion of the amplified PCR product with BamHI and XhoI restriction enzymes, ligation into pENTR2B, and then Gateway cloning the fragment into the modified pH7WGY2 vector. *Agrobacterium*‐mediated transformation of Brachypodium callus and transgenic plants generation were as previously described (Cass *et al*., 2015; Petrik *et al*., 2014).

### *AsFMT*‐expressing plant identification and genotyping

Transgenic *EYFP:AsFMT*, *AsFMT:EYFP* and *EYFP:GUSPlus* plants were identified and confirmed by seedling selection on hygromycin (Petrik *et al*., 2014), by tracking EYFP fluorescence emanating from the EYFP:AsFMT or AsFMT:EYFP fusion proteins using a Leica MZ8 fluorescence dissecting scope, by Western blot analysis, and/or by PCR amplification of portions of the constructs. For PCR analyses, leaf genomic DNA was extracted with the ExtractNAmp Plant PCR kit (Millipore Sigma, St. Louis, MO, USA) and used as a template. The fusion proteins in *EYFP:AsFMT‐*, *AsFMT:EYFP‐* and *EYFP:GUSPlus‐*expressing plants were assessed by Western blot analysis of stem tissue protein extracts detected with an anti‐GFP (also detects EYFP) antibody (1:2500 dilution, Allele Biotechnology and Pharmaceuticals Inc., San Diego, CA, USA catalog # ABG‐MP‐MMGFP10; secondary antibody: 1:5000 goat anti‐mouse IgG‐HRP, Thermo Fisher Scientific, Waltham, MA USA catalog # 31430).

### Generation of *Bdccr1‐1* plants and lignin imaging

A T_2_‐generation seed stock (lot JJ8708) harbouring a T‐DNA insertion in Bradi3g36887 located at Chr3 Bd3:38994367… 38999617 forward was obtained from Dr. John Vogel and the Western Regional Research Center (Bragg *et al*., 2012). This allele, designated *Bdccr1‐1*, harboured the pJJ2LBA‐derived T‐DNA activation tagging construct in the third intron of *BdCCR1* at position Bd3:38996225..38996226 (Phytozome v. 12.0, https:// phytozome.jgi.doe.gov/pz/portal.html). Tracking of the *Bdccr1‐1* T‐DNA insertion was carried out using standard PCR and the following primer pair: forward 5′ ‐ACGCAATTTCTGGATGCCGACAG‐3′ and reverse 5′‐CTAATGTCAAAGTCGATGGTACGAAGC‐3′ (578 bp PCR product). PCR analysis showed the T‐DNA activation tagging insertion co‐segregated with the described phenotypes, which, like *ccr* mutations in other species, was recessive. Phloroglucinol‐HCl staining of *Bdccr1‐1* and corresponding wild‐type plants was performed as described in (Petrik *et al*., 2014).

### CCR1 expression analysis

Total RNA was extracted from culm plus leaf sheath tissue of the apical internode from plants grown 35–45 days in soil by first pulverizing the tissue in liquid nitrogen, and then isolating the RNA using a Plant RNeasy RNA extraction kit following manufacturer’s directions (Qiagen, Germantown, MD, USA). 0.5 µg DNaseI‐treated (NEB) RNA was reverse transcribed using oligo(dT)_18_ and M‐MLV (Promega, Madison, WI, USA) in a 20 μL reaction volume. First‐strand cDNA samples were used as PCR templates by dilution to a final concentration of 5 ng/μL in a 20 μL reaction volume. The following primer pairs, expected PCR product size, and thermal cycling conditions were used for Reverse Transcription‐semiquantitative PCR assessment of *BdCCR1* (Bradi3g36887) transcript levels. Upstream of the insertion site (pair F1‐R1), the primers were forward 5′‐GAGAATCCTACCAAACGTTACCAACTCG‐3′, and reverse 5′‐ACGACATCGACCACGGTCATCTTG‐3′, 144 bp; and primers downstream of the insertion site (pair F2‐R2) were forward 5′‐GTGGCGTAACCATCCGAGCATG‐3′, reverse 5′‐AAATCCACTTCTGAACATTAGCAACCG‐3′, 157 bp. For amplification of the reference gene, *EF1α* (Bradi1g06861, that used to be locus Bradi1g06860 in Hong *et al*., 2008) the primers were forward 5′‐TCACCATCGATATTGCCTTGTGGAAG‐3′, and reverse 5′‐GTCTGGCCATCCTTGGAGATACCAG‐3′, 196 bp. Thermal cycling conditions used: initial dissociation, 95 °C for 2 min; then 33 cycles of 95 °C for 20 s, 56 °C for 20 s, 72 °C for 20 s; followed by a final extension of 95 °C for 5 min.

### Generation of *Bdpmt* mutant plants

The Brachypodium *Bdpmt* mutant (in which guanine was replaced by adenine at position 563 of the Bradi2g36910 ORF) was generated in a sodium azide‐mutagenized population generated by the Institut National de la Recherche Agronomique (INRA; Dalmais *et al*., 2013) and characterized in previous manuscripts (Petrik *et al*., 2014).

### Cross‐pollination and genotyping stacked‐trait plants

Brachypodium plant lines were cross‐pollinated as described in an online illustrated protocol (Steinwand and Vogel).

### *AsFMT:EYFP × Bdpmt‐1* plant identification

The *Bdpmt‐1* mutation was confirmed by tracking the mutation with a Cleaved Amplified Polymorphic Sequence (CAPS) marker using primers Bdpmt‐1_CAPS_F 5′‐ TGCCGGCATTTTGCACTTGTAGATTCTGC‐3′ and Bdpmt‐1_CAPS_R 5′‐ AGCGGTGGCCCGTGGAGGCGAAGTA‐3′ (742 bp PCR product; Wild‐type NarI digestion: 521 bp, 111 bp and 110 bp fragments; *Bdpmt‐1* NarI digestion: 631 bp and 111 bp fragments). The AsFMT:EYFP was confirmed by tracking the EYFP fluorescence using a Leica MZ8 fluorescence dissecting scope.

### Plant tissue preparation for chemical analysis

Senesced culm tissue was air dried, ground to a fine powder, and then solvent‐extracted sequentially with water (3 × 45 mL), 80% ethanol (3 × 45 mL) and acetone (2 × 45 mL), by first suspending the sample in solvent, sonicating for 20 min, pelleting by centrifugation (8800 ***g***, 20 min, Sorvall Biofuge primo centrifuge), and finally decanting the supernatant. The extract‐free pellet was then dried under vacuum prior to analysis. We refer the resulting dry powder as alcohol‐insoluble residue (AIR), even though additional solvents were used to ensure complete removal of extractives.

### DFRC procedure

The DFRC method was performed as described previously for the quantification of ML‐FA conjugates and tricin using diethyl 5‐5′‐diferulate diacetate as the internal standard (Karlen *et al*., 2016; Lan *et al*., 2016). The DFRC assay results are listed in Table 1. Significance was determined from three biological replicates of each sample with a Student’s *t*‐test with *P* < 0.05 or 0.01.

### Procedure for determining acetyl‐bromide‐soluble lignin (ABSL) content

The ABSL contents were measured in 1 cm quartz cuvettes on a Shimadzu UV‐1800 at λ = 280 nm and ɛ_280_ = 20.0, as previously described (Fukushima and Hatfield, 2001; Hatfield *et al*., 1999). The ABSL assay results are listed in Table 1.

### Klason lignin of *Bdccr1‐1* plants

Klason lignin was determined using a modified Klason lignin analysis method, as previously described (Cullis *et al*., 2004).

### Thioacidolysis of *Bdccr1‐1* plants

The thioacidolysis protocol was performed as previously described (Bouvier d’Yvoire *et al*., 2013). Lignin‐derived thioacidolysis monomers were identified by GC‐MS as their trimethylsilylated derivatives.

### Partial saccharification analysis

Partial saccharification analysis was performed as described in (Santoro *et al*., 2010). Samples were pretreated with 1 mm or 6 mm NaOH prior to measuring glucose and pentose release. Significance was determined from three biological replicates of each sample using a Student’s *t*‐test (*P* < 0.05).

## Competing interests

The authors declare the following competing financial interest: J. Ralph (Feruloyl‐CoA:Monolignol Transferase US2013/0203973) and, as a holder of this patent, may benefit financially from advances in the technology discussed in the paper.

## Author contributions

JR, JS, CC and SK conceived of the experiments and interpreted data. CC, RAS, DLP, DP and SK carried out the experiments and analysed the data. RAS, CC, JS and SK wrote the paper. All authors edited and approved the final manuscript.

## Supporting information

**Figure S1** Confirming gene expression, and production of an active YFP protein.
**Figure S2** Mature *AsFMT:EYFP* expressing transgenic Brachypodium plants grown side‐by‐side with wild‐type and *Bdpmt‐1* knockout mutant plants.
**Figure S3** Characterization of the *Bdccr1‐1* T‐DNA mutant.
**Figure S4** Characterization of the *Bdccr1‐1* T‐DNA mutant.

## Data Availability

The data that support the findings of this study are available from the corresponding author upon reasonable request.
