# Supplementary material for: Stacking AsFMT overexpression with BdPMT loss of function enhances monolignol ferulate production in Brachypodium distachyon
Source: Plant Biotechnol J. 2021 May 15;19(9):1878–86. doi: 10.1111/pbi.13606 (PMC8428837; doi:10.1111/pbi.13606)
Supplement: Supplementary file 1 — Figure S1 Confirming gene expression, and production of an active YFP protein. Figure S2 Mature AsFMT:EYFP expressing transgenic Brachypodium plants grown side‐by‐side with wild‐type and Bdpmt‐1 knockout mutant plants. Figure S3 Characterization of the Bdccr1‐1 T‐DNA mutant. Figure S4 Characterization of the Bdccr1‐1 T‐DNA mutant. [file PBI-19-1878-s001.pdf]

Stacking *AsFMT* overexpression with a *BdPMT* loss of function enhances monolignol ferulate production in *Brachypodium distachyon*

Rebecca A. Smith, Cynthia L. Cass, Deborah L. Petrik, Dharshana Padmakshan, John Ralph, John C. Sedbrook, and Steven D. Karlen

SUPPLEMENTAL DATA

Figure S1. Confirming gene expression, and production of an active YFP protein.

Figure S2. Mature *AsFMT:EYFP* expressing transgenic Brachypodium plants grown side-by-side with wild-type and *Bdpmt-1* knockout mutant plants.

Figure S3. Characterization of the *Bdccr1-1* T-DNA mutant.

Figure S4. Characterization of the *Bdccr1-1* T-DNA mutant.

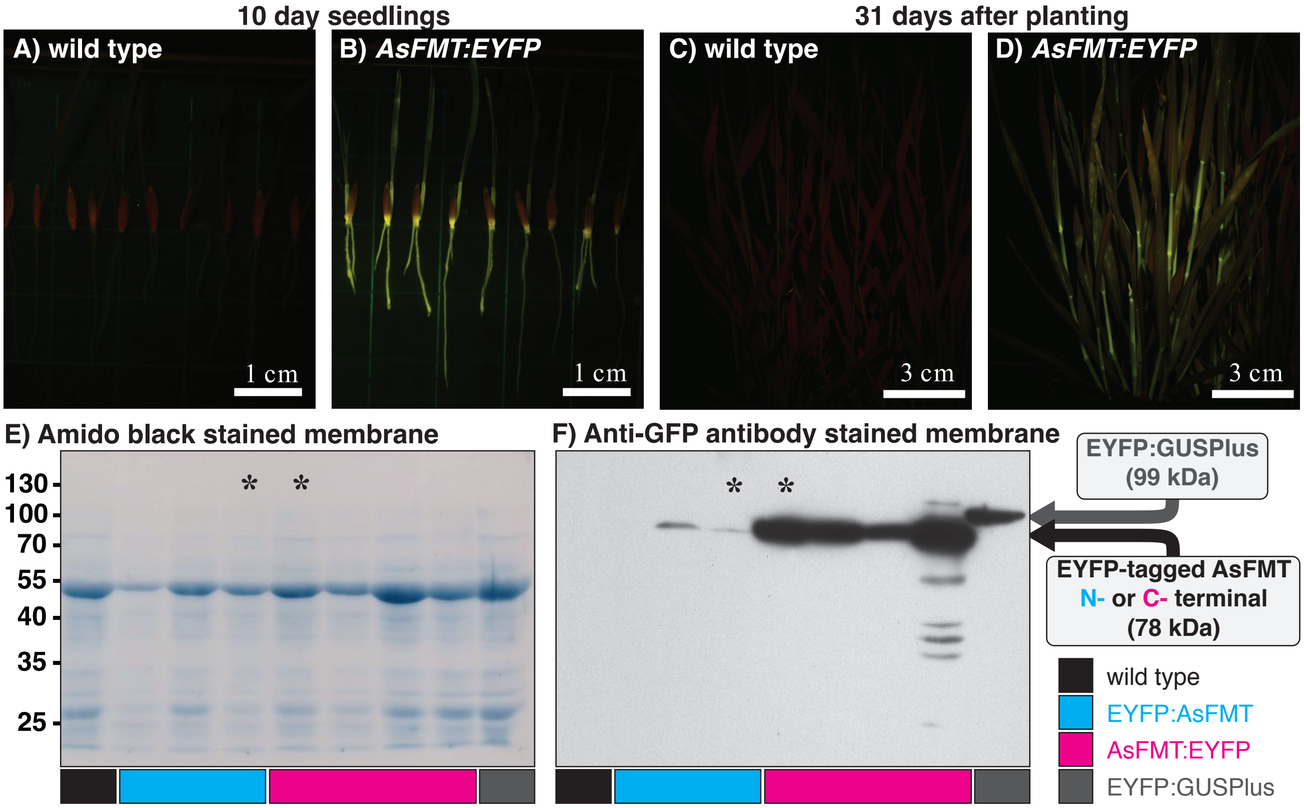

Figure S1. Confirming gene expression, and production of an active YFP protein. Images of *YFP* fluorescence of *AsFMT:EYFP* seedlings (A,B) and mature plants (C,D). The wild-type plants show no YFP fluorescence as either seedlings (A) or 31 days after planting (C), whereas the *AsFMT:EYFP* seedlings strongly fluoresce yellow, indicating expression and production of an active YFP protein. Bottom: Representative Western blot analysis of proteins extracted from independent event T_3-generation plant leaves. (E) Amido black stained membrane showing protein loading. (F) Leaf proteins probed with anti-GFP antibody, 15 s exposure. AsFMT:EYFP and EYFP:AsFMT expected band size: 78 kDa; EYFP:GUSPlus expected band size: 99 kDa (arrows). WT stands for wild type. Asterisks indicate lines tested by DFRC for cell-wall-bound ML-FA.

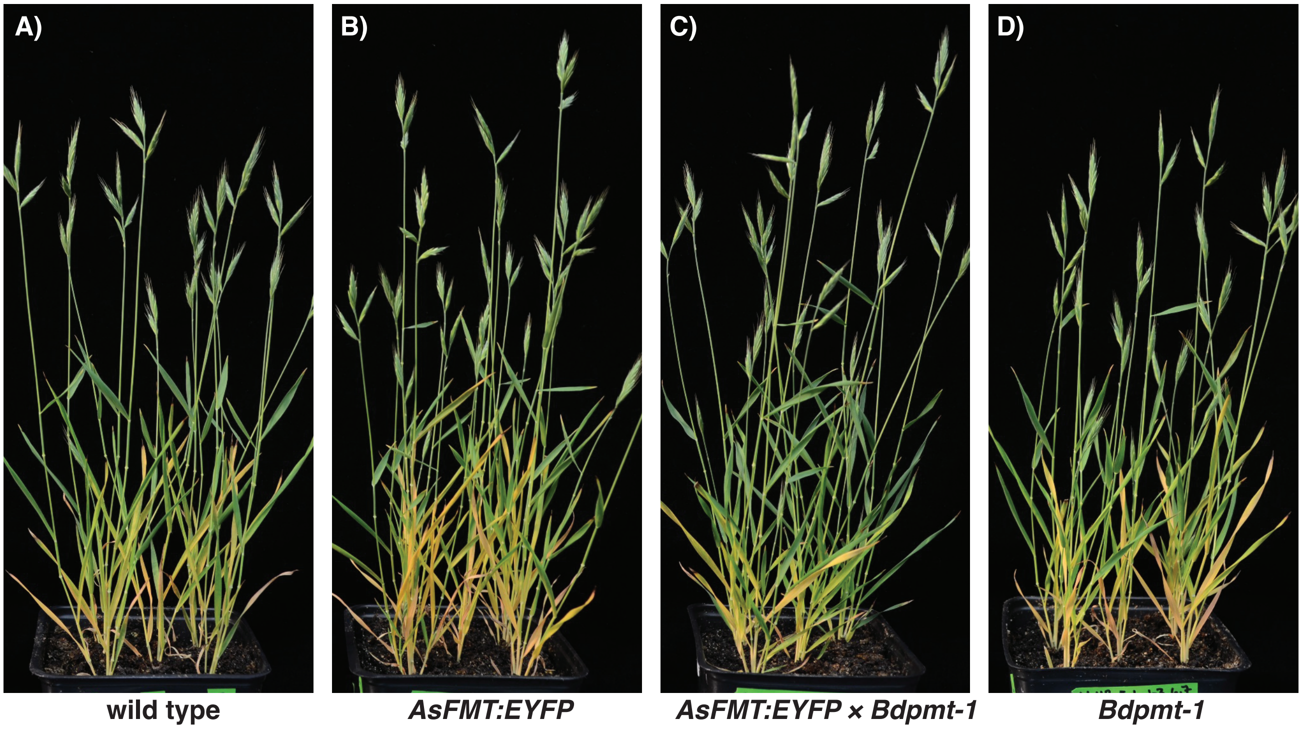

Figure S2. Mature *AsFMT:EYFP* expressing transgenic Brachypodium plants grown side-by-side with wild-type and *Bdpmt-1* knockout mutant plants. From left to right (A) wild type, (B) *AsFMT:EYFP*, (C) *AsFMT:EYFP* × *Bdpmt-1*, and (D) *Bdpmt-1*.

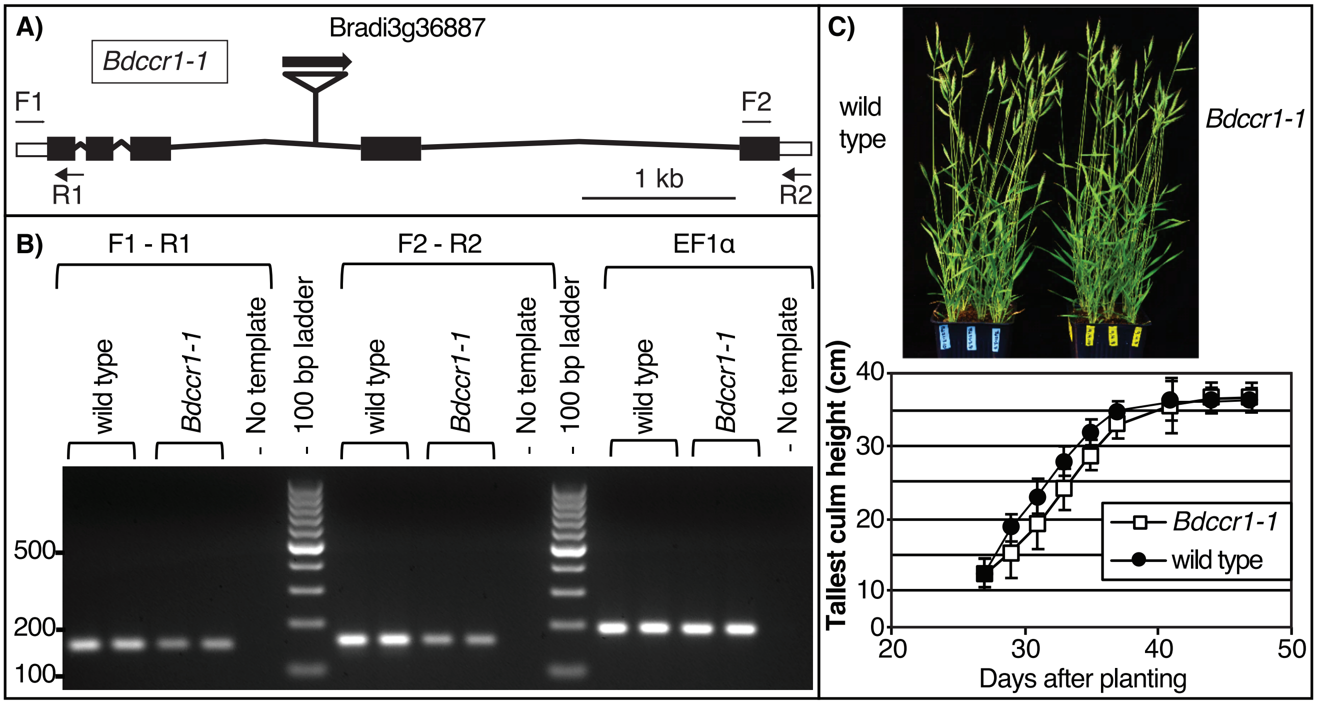

Figure S3. Characterization of the *Bdccr1-1* T-DNA mutant. (A) Scale diagram of the *BdCCR1* locus, Bradi3g36887. Black and white boxes represent exons and untranslated regions, respectively, and lines indicate introns. The black triangle represents the location of the pJJ2LBA T-DNA insertion (not shown to scale) in seed lot JJ8708 (*Bdccr1-1*) along with relative primer locations (arrows). (B) Agarose gel-electrophoresed PCR products semi-quantitatively amplified from first strand cDNA indicating the amounts of transcript present either upstream (primers F1 - R1, 144 bp) or downstream (primers F2 - R2, 157 bp) of the T-DNA insertion. Note the fainter *Bdccr1-1* bands signifying a partial reduction in *BdCCR1* transcript levels. EF1α (Bradi1g06860) is the loading control (196 bp). Ladder = ThermoScientific 100 bp SM0421. (C) *Bdccr1-1* plants grown in 4”-diameter pots appear indistinguishable from wild type, having a slight yet insignificant delay in growth as determined by daily culm height measurements (n=15, bars are std. dev.).

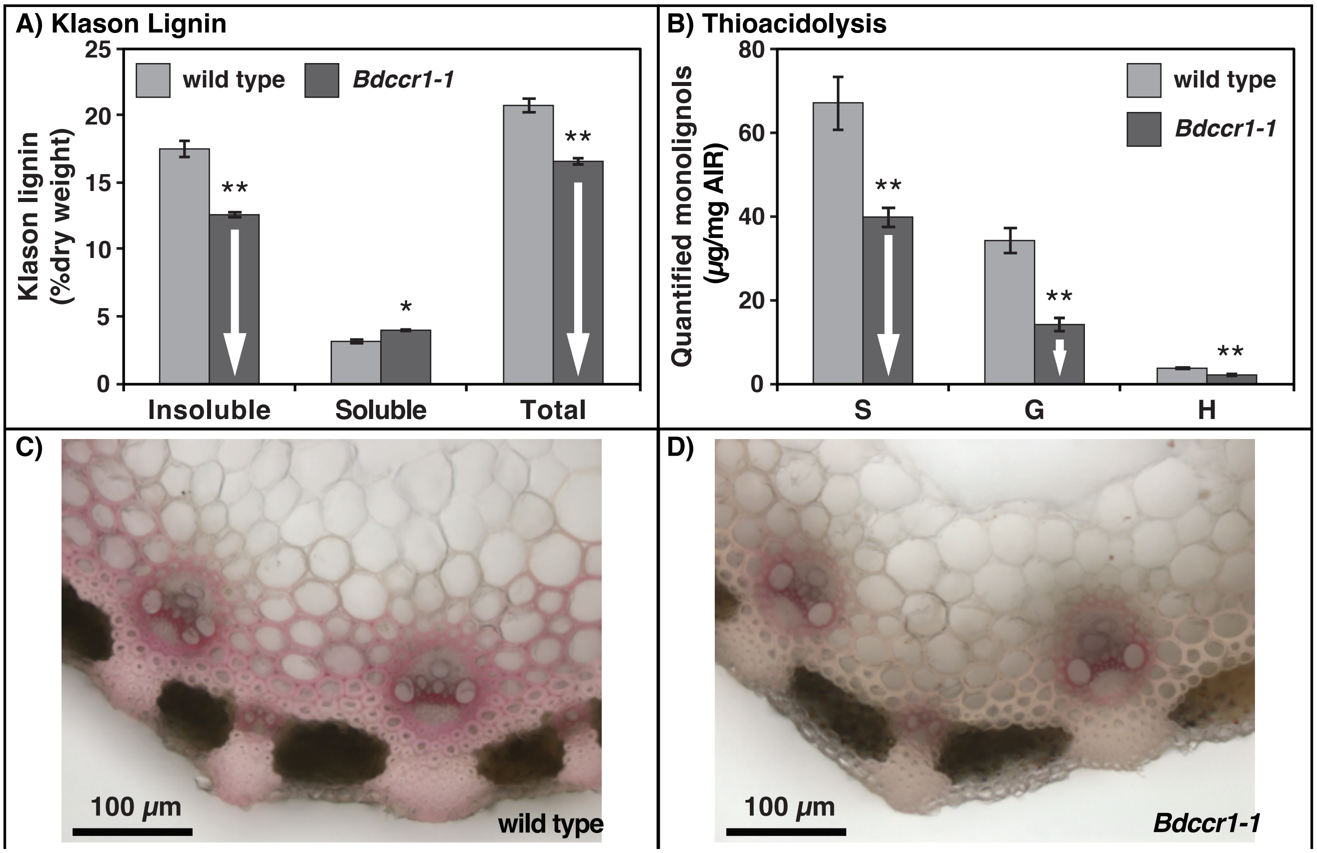

Figure S4. Characterization of the *Bdccr1-1* T-DNA mutant. (A) Klason Lignin quantification of senesced stems plus leaf sheaths of *Bdccr1-1* and wild-type Bd21-3. Each biological rep bar represents the mean of three technical reps. (B) Thioacidolysis quantification of Syringyl (S), Guaiacyl (G), and *p*-Hydroxyphenyl (H) lignin units. Bars indicate SEM for n = 3-5 plants. Significant differences from wild type, as determined by the Student's t-test, are indicated in plots (A, B) with * for $\rho<0.05$ and ** for $\rho<0.01$. (C, D) Phloroglucinol staining of transverse stem sections of wild type (C) and *Bdccr1-1* (D) taken from plants at similar developmental stages: Scale bar = 100 µm.
